# Boosting COVID-19 vaccine inoculation and booster shots: a systematic review and meta-analysis of factors that influence Coronavirus vaccine uptake in practice

**DOI:** 10.4314/ahs.v23i2.3

**Published:** 2023-06

**Authors:** Frank Adusei-Mensah, Nzechukwu Michael Isiozor, David Lekpa Kingdom, Kennedy Jeff Oduro, Chukwuma John Okolie

**Affiliations:** 1 Institute of Public Health and Clinical Nutrition, University of Eastern Finland, Kuopio, Finland; 2 Public Health and Medicine Research Group, Center for Multidisciplinary Research and Innovation, Jyvaskyla, Finland; 3 Institute of Clinical Medicine, Faculty of Health Sciences, University of Eastern Finland, Kuopio, Finland; 4 Department of Anatomy, Faculty of Basic Medical Sciences, College of Health Sciences, University of Port Harcourt, Rivers State, Nigeria; 5 Department of Medicine and Therapeutics, Cape Coast Teaching Hospital, Cape Coast, Ghana; 6 Department of Surveying and Geoinformatics, Faculty of Engineering, University of Lagos, Nigeria

**Keywords:** Vaccination, vaccine hesitancy, coronavirus, pandemic, herd immunity, immunization

## Abstract

**Introduction:**

Vaccines alone do not control pandemics, but vaccinations. The hope of COVID-19 pandemic control is hinged on vaccinations and other public health measures. This systematic review/meta-analysis (SR/MA) investigated the factors that inform coronavirus vaccine uptake globally in an attempt to improve COVID-19 immunization.

**Method:**

The PRISMA 2020 methodology was used for this review. A total of 2902 articles were identified from electronic databases and other sources. After screening, 33 articles were included in the review and quantitative meta-analysis. Comprehensive meta-analysis software version 3 was used for the meta-analysis.

**Results:**

We observed that vaccine effectiveness, side effects and the proportion of acquaintances vaccinated significantly influenced respondents' COVID-19 immunization decision. Also, associations of vaccine effectiveness, smaller risks to serious side effects, free and voluntary vaccinations and fewer vaccine doses, and longer duration to wanning were observed. We also observed variations in vaccine hesitancy trends in studies carried out in Asia, Europe, America, and Africa.

**Conclusion:**

Wanning and acquaintance's vaccination status as factors to vaccination are insights the present paper is bringing to the limelight. Health promotion and COVID-19 vaccination planning are crucial for enhancing vaccine uptake.

## Introduction

The coronavirus pandemic has rampaged the world with tens of thousands infected daily and thousands of deaths reported every day. Due to the continual spread of the disease, the eyes of many stakeholders are fixed on vaccination with the approved vaccines for the control of the pandemic. Despite the rigor and cost implications of vaccine development, their impact can only be realized upon a successive roll-out of the available vaccines. However, vaccine uptake is not without challenges and more so with the current pandemic. It is therefore crucial for proper understanding and identification of these bottlenecks by stakeholders to successively plan and improve coronavirus vaccine uptake with regards to geographical, professional, age and sex differences. Some challenges of vaccination including side effects, inefficacy and lack of motivation are known. However, the fast rate of coronavirus vaccine development, the use of Messenger Ribosomal Nucleic Acid (mRNA) approaches, the non-existence of long-term follow-up before global roll-out, and the presence of multiple producers with varying reported vaccine efficacies exacerbate the bottlenecks of coronavirus vaccine uptake, and this requires urgent research. To aid researchers and policymakers in planning and improvement of coronavirus vaccine uptake, this review provides answers to the question: what factors influence coronavirus vaccine uptake in clinical practice? The main aim is to evaluate an array of factors that inform coronavirus vaccine uptake and that could be considered in the improvement of vaccination among various groups and regions and for efficient control of the pandemic.

## Background

Coronaviruses were first found in humans in the 1960s and have been known to cause mild respiratory tract infections^1^. This class of viruses are zoonotic, and have been isolated in several animals, but typically, bats are accepted as their natural habitat^1^. In humans, coronaviruses are amongst the common causes of the common cold. However, recently detected viruses in this family such as the Severe Acute Respiratory Syndrome Corona Virus 2 (SARS-CoV-2, 2002), Middle Eastern Respiratory Syndrome Coronavirus (MERS-CoV, 2012), and the COVID-19 viral infection have completely modified current approaches to the group of viruses as they tend to cause severe acute respiratory diseases requiring hospitalization and intensive care^2^,^3^,^4^.

The SARS–CoV-2 virus has infected hundreds of millions of people across all races and age groups leading to the deaths of over 3 million humans. The viral infection has been found to have a predilection for human-to-human transmission as the incidence of the disease is higher in crowded populations warranting the need for interventions that reduce human-to-human contact to the barest minimum to be introduced^1^. This requires some lifestyle modifications which makes adherence and effectiveness difficult, unsustainable and unpredictable, thus creating the need for an alternative that can reduce the incidence and severity of the disease, and associated mortalities - a vaccine.

In December 2020, the COVID-19 vaccine from Pfizer/ BioNTech was accepted for emergency use in the United States (US) by the Food and Drug Administration (US FDA)^5^. After this announcement, many large pharmaceutical companies from various countries have in turn also received this approval, and have begun mass distribution of their vaccines around the world. The vaccines currently in use around the world include Pfizer/BioNTech, Moderna (Cambridge and Massachusetts), Oxford-AstraZeneca vaccine, Sputnik V (Russia), and many more. There are currently over 58 vaccines that have been developed and are at different phases of clinical trials^6^. The vaccines currently in use vary in their manufacturing, part of virus used, mechanism of action and consequently, the efficacy. The Moderna and Pfizer vaccines both developed in the US are mRNA vaccines and have a reported efficacy of 94 - 95%. The Chimpanzee adenovirus-vectored AstraZeneca vaccine achieved an efficacy of 62% in early clinical trials^7^. Other adenovirus-based vaccines such as the Russian Sputnik V and the Johnson and Johnson (J&J)/Janssen have preliminary efficacy reports of 92% and 72% respectively^6^.

**Figure 1:**
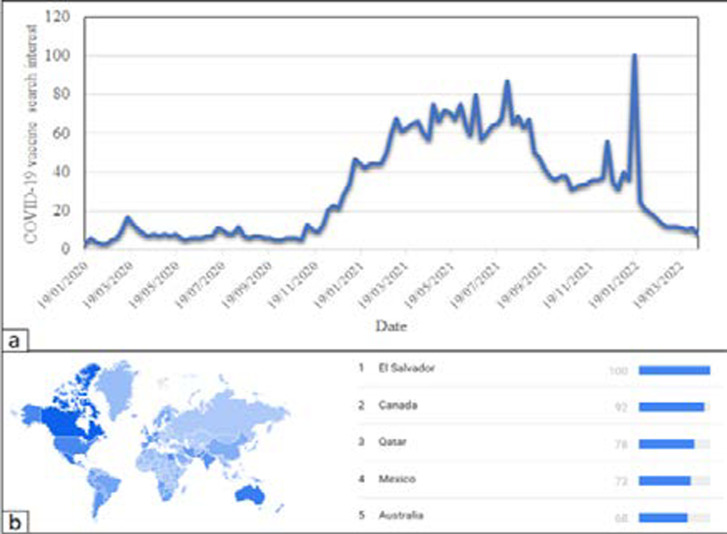
Global awareness of COVID-19 vaccine. The graph (1a) shows fluctuations in the global search interest in the COVID-19 vaccine from January 2020 to April 2022. Figure 1b shows the distribution of countries based on awareness level, and the top-5 countries with high awareness of the COVID-19 vaccine based on internet searches (Generated from Google Trends on 13th April 2022: search term ‘COVID 19 vaccine’)

The general awareness of the COVID-19 vaccines being rolled out in different parts of the world has been increasing. Figure 1 presents a graph generated using Google Trends data showing the worldwide interest in the COVID-19 vaccine between January 2020 and April 2022. Google Trends provides access to a large sample of unfiltered, anonymized and aggregated search requests made by internet users on Google thus enabling the analysis of worldwide interest in a particular topic 8. In the graph, the numbers ranging from 0 to 100 represent the worldwide search interest relative to the highest point on the chart for the given period. From November 2020 onwards when the deployment of the vaccine in several countries was imminent, there was an increased global interest in the COVID-19 vaccine. This could be attributed to several interventions. For example, in December 2020, the United Kingdom was the first country in the world to deploy an approved COVID-19 vaccine 9. After several vaccines were deployed, there were aggressive awareness campaigns in many countries to encourage their citizens to take the vaccine. These publicity programs and other unspecified incidents or interventions account for the observed increases, sudden accelerations (spikes), and lulls in the global interest to date. Between 2020 and 2021, there was relatively high interest in the vaccines, with the peak popularity occurring between March and August 2021. It is pertinent to note that a high-level vaccine awareness does not guarantee an elevated level of vaccine uptake.

While it is not straightforward to compare the efficacies of various vaccines due to the fact that the studies were done at different times, in different populations, and geographical locations with varying population characteristics, the European Medicines Agency (EMA) and the US FDA have set a cut-off of 50% as the efficacy required for a COVID-19 vaccine approval 6. Aiming for a 75% population vaccine coverage, the required efficacy to prevent an epidemic is 70% and the efficacy required to do so without additional measures such as social distancing is 80%^10^.

Vaccine rollouts have begun in many countries across the globe. Many challenges mitigate against successful vaccine uptake in the general population. This can slow progress in achieving herd immunity. There is a need to identify challenges early in the roll-out phase, including the factors that can hinder successful large-scale vaccine uptake among the general population.

The research question framework for this review is based on the PICO framework as defined below:

- Population (P) - Global population
- Intervention (I) - Willingness to receive COVID-19 vaccine.
- Comparison (C) - Non-willingness to receive COVID-19 vaccine.
- Observation (O) - Improved vaccination and booster shots in practice.

## Review Methodology

**Figure 2:**
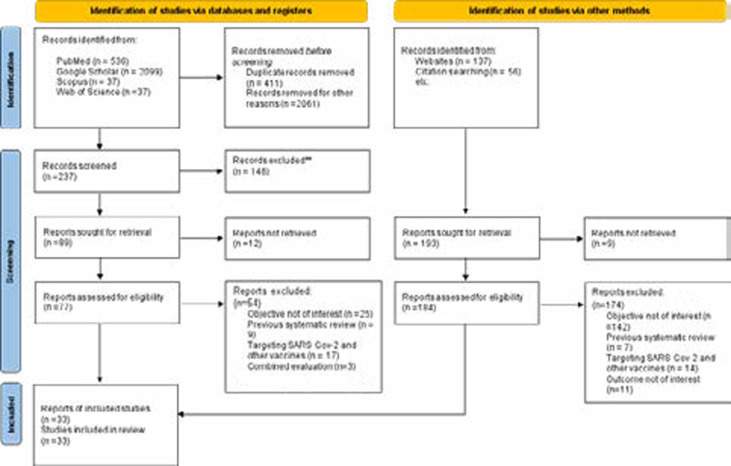
PRISMA 2020 flowchart for identification and inclusion of articles in the systematic review and meta-analysis

The Preferred Reporting Items for Systematic Reviews and Meta-Analyses - PRISMA 2020 guidelines were adopted for this review^11^. More information on the PRISMA statement is available on the website (http://www.prisma-statement.org/). There are 3 stages in the PRISMA workflow: identification, screening and inclusion. The PRISMA flow diagram for this review is presented in Figure 2.

### Eligibility Criteria

For studies to be included, the publication language must be English, the survey must be carried out between 1st November 2019 to 15th February 2021, the studies should be performed on human participants, the aim(s) of the study must relate to finding the participants willingness or hesitance to receive COVID-19 vaccines, and the study should be a published article with a journal or a DOI indexed preprint. Papers evaluating the willingness to receive COVID-19 vaccines and other vaccines in a single study were excluded. In addition, studies not meeting the above inclusion criteria were excluded. Summarily, the following criteria were applied to select relevant articles:

#### Inclusions

1. The publication language must be English.
2. The survey must be carried out between 1st November 2019 to 15th February 2021.
3. The studies should be performed on human participants.
4. The aim(s) of the study must relate to finding the participants' willingness or hesitance to receive the COVID-19 vaccine.
5. The study should be a published article in a journal or a DOI-indexed preprint.

#### Exclusions

1. Studies not related to vaccine uptake.
2. Studies on vaccine efficacy or mechanistic studies.
3. Studies on vaccines other than SARS-CoV-2.
4. Predictive studies conducted on models other than humans.
5. Studies targeting COVID-19 and other vaccines.
6. Previously published systematic reviews on SARS-CoV-2.
7. Studies on COVID-19 vaccine trials.

### Search Strategy

For the search, Medical Subject Headings (MeSH) terms were generated from the MeSH library. A comprehensive literature search was performed in four different databases namely Google Scholar, PubMed, Web of Science and Scopus. Several MeSH terms including ‘COVID-19’, ‘SARS-CoV-2’, ‘vaccine’, ‘inoculation’, ‘immunization’, ‘willingness’, ‘hesitancy’, ‘factors’, ‘reasons’, and ‘factors associated’ were integrated using Boolean operators (AND/OR).

### Identification of studies and screening

A total of 2902 articles were identified from electronic databases (PubMed, Google Scholar, Web of Science and Scopus), and website and citation searches (Figure 2). Extracted records were saved with the first author's surname and the year of publication into a Microsoft excel spreadsheet for further consideration. After removing duplicates (n = 411), the remaining records were screened for titles and abstracts. Summarily, 77 peer-reviewed articles were eligible for further examination and finally, 33 articles were considered for the qualitative systematic review and quantitative meta-analysis. The main features of the selection criteria and included studies are presented in Figure 2 and Table 1.

**Table 1:** Characteristics of selected studies and COVID-19 vaccine acceptance rates

Continent	Study	Country	Methodology	Survey Date	Response for Vaccine Acceptance	N	Target Population	Acceptance Rate (%)	Gender Distribution. Male (M), Female (F)
Asia	12	China	SRS	2020	Agree to vaccinate	1883	GP	45.82%	NA
	13	Hong Kong	Random telephone survey	July - Aug. 2020	Acceptance	1200	GP	37.2%	M = 28.7% F = 71.3%
	14	Bangladesh	E-survey	Dec. 2020 - Feb. 2021	Agree	1658	GP	58.6%	F = 59% (434) M = 58.2% (537)
	15	Kuwait	Web-based C-S	Aug. - Sept. 2020	Definitely and probably	2368	GAP	53.1%	M = 58.3% (434/744) F = 50.9% (812/1597)
	16	China	O. CS	May - June 2020	Yes	3195	GAP	83.8%	M = 85.2% (1163) F = 82.9% (2032)
	17	Jordan	Web-based survey	-	Yes	1144	Middle Eastern Population	36.8	NA
	18	China	OS	Oct. -Nov. 2020	Yes	1009	GAP	60.4% (609)	M = 38.8% F = 61.2%
	19	Hong Kong, China	Telephone survey	Sept. 2020	Yes, soonest	450	GAP	81.3%	NA
	20	Jordan, Kuwait and Saudi Arabia	OS	Dec. 2020,	Yes	3414	GAP	29.4%.	M = 430 (38.6) F = 550 (23.9)
	21	China	OS	Nov. 2020	Intention to receive	6922	University students	78.9%	NA
North America	22	US	O. and internet survey	June 2020	Extremely or somewhat likely	804	GP	62.2%	M = 71.9% (373) F = 53.8% (431)
	23	US	C-S, OS	April - May 2020	Very and extremely willing	486	Multiple sclerosis adults	66.0%	NA
	24	US	App-based survey	Nov. 2020	Somewhat and very willing	7402	Aged population (>65 yrs) with	91.3%	F = 87.9% (3423) M = 94.3% (3979)
	25	US	Mobile phone, OS	Dec. 2020	Very likely	2650	GAP	40%	F = 75% (1213) M = 85% (1417)
	26	US	Multicenter cohort study	Nov. - Dec. 2020	Likely and definitely	2135	HCW	69%	NA
	27	US	CS	Aug. - Dec. 2020	Yes	948	GP	28.1%	M = 124 (39.9%) F = 141 (26.2%)
	28	US	Institutional email list survey	Nov. - Dec. 2020	Agree and strongly agree	5287	HCP	57.5	M = 992/ 1339 (72.5%) F = 013/3842 (52.4%)
	29	US	Web and telephone	May 2020	Yes	1043	GAP	53.60%	F = 355/731 (63.5%) M = 204/312 (65-38%)
	30	US	OS	June 2020	Very and somewhat likely	1878	GAP	78%	M = 709/910 (78) F = 758/968 (78%)
	31	Philadelphia, US	Electronic survey	Nov. - Dec. 2020	Planning to Receive	12034	Hospital employees	63.7%	M = 81.7% (2064/ 2525) F = 5181/ 8622 (60.1%)
	32	US	O. snowball. S	Oct. - Nov. 2020	Yes	3479	HCWs	36%	F = 818 (31%) M = 425 (49%)
Europe	33	UK	Stratified, OS	Sep. - Oct. 2020.	Very likely	32361	GP	63.5%	NA
	34	UK	Clustered-stratified O. S	Nov.-Dec. 2020	Likely and very likely.	9956	Household Longitudinal	82%	M = 14.7% (4666) F = 21% (5290)
	35	Greece, Albania, Cyprus, Spain, Italy, Czech Republic, Kosovo	Web survey	Dec. 2020	Somewhat and completely agree	2249	nursing students	43.8%	F = 1902 M = 344
	36	UK	C-S surveillance	Feb. 2021	Willing	12278	HCW	64.5%	F = 75.7% M = 24.3%
	37	France, Canada, Belgium	Questionnaire survey	Oct. - Nov. 2020	Yes, certainly/probably	2,678	HCWs	71.62	NA
	38	Romania and Bulgaria	OS	July - Aug. 2020.	Agree	395	Community Pharmacists	50%	NA
	39	Greek	CAT and web Interview	April - May, 2020	Yes	1004	GAP	57.7%	M = 308 (60.1%) F = 271 (55.1%)
	40	Ireland UK	Quota S.	March 2020	Accepting	Ireland 1041) UK = 2025	GAP	Ireland = 64.9% UK = 67%	NA
	41	Spanish territory	O. Twitter survey	Sept. -Nov. 2020	Acceptance	731	GP with twitter	77.6%	M = 332 (79.2%) F = 405 (76.5%)
Africa	42	Egypt	C-S, OS	Dec. 2020 - Jan. 2021	Somewhat and totally agree	488	health care employees	45.9	NA
	43	DRC	OS	Aug. - Sept. 2020	Yes	4131	GP	2310 (55.9%)	NA
	44	Cameroon	OS	May - Aug. 2020	Non-hesitant	2512	GP	15.4	NA

**Key:** OS = online survey, GP = general population, GAP = general adult population, HCW = healthcare workers, HCP = healthcare personnel, SRS = stratified random sampling, CAT = computer assisted telephone, DRC= Democratic Republic of Congo, US = United States, UK = United Kingdom, C-S = cross-sectional, CS = convenience sample, O = online, S = sample.

### Data Extraction

The year of publication, the first author's surname, the date of the study, the country of study, the number of participants and the information on the participants' willingness to accept SARS-CoV-2 vaccines were extracted from the selected studies and compiled in a Microsoft Excel spreadsheet. The summary of the extracted data is presented in Table 1.

### Quantitative Synthesis and Reporting

The comprehensive meta-analysis software version 3 released by Biostat Inc. was used for the meta-analysis. The pooled effect was estimated with a forest plot, and the potential impact of publication bias was assessed using the funnel plot. The quality of the analysis was assessed using Cochrane's Q2 and tau square (I^2^) estimations.

## Results

### Characteristics of selected studies

**Figure 3:**
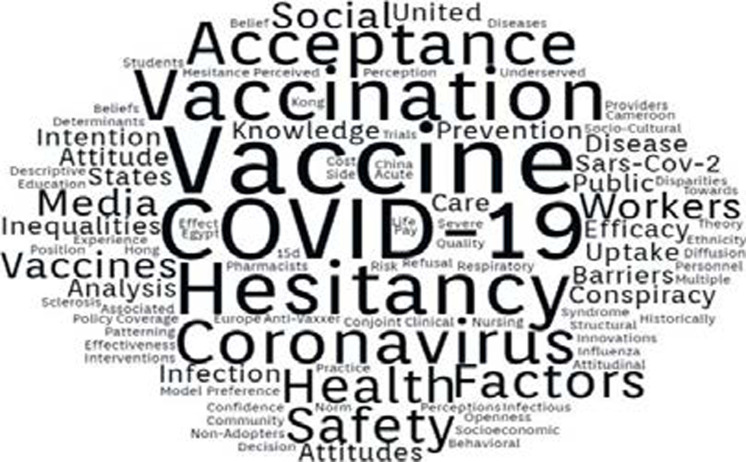
Word cloud showing the most frequent terms in the keywords of included studies

Figure 3 shows the word cloud for this review, a visualization of the highest occurring terms/expressions in the keywords of the included studies. The sizes of the font are directly proportional to the frequency of occurrence of the terms shown. From this figure, it appears that vaccine hesitancy has been a source of concern and an issue of interest to researchers. Other factors that could influence vaccine uptake are captured by keywords such as ‘inequalities’, ‘attitudes’, ‘efficacy’, ‘conspiracy’, ‘intention’, ‘barriers and ‘knowledge’. It also appears that there was less emphasis in the literature on ‘perceptions’ and ‘beliefs’ and these are factors that also influence vaccine uptake.

Table 1 represents a summary of the characteristics of the selected studies. Most identified studies were carried out in developed countries. The willingness to COVID-19 vaccine uptake was seen to vary across regions with Russia and some Middle Eastern and African countries reporting low willingness to take the SARS-CoV-2 vaccines.

### Geographic disparities in acceptance rates

**Figure 4:**
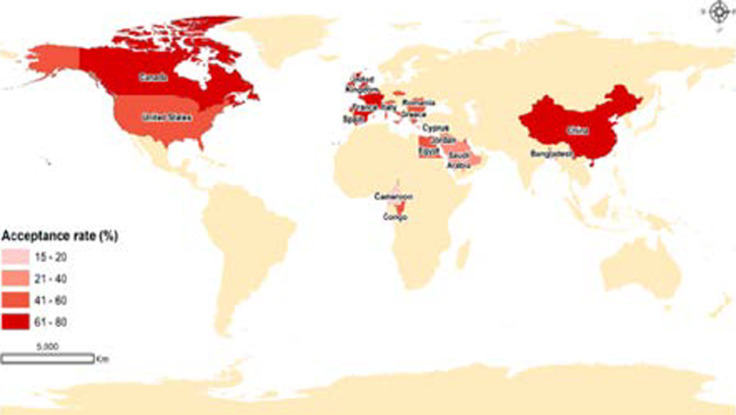
World map displaying the geographic distribution of acceptance rates

Figure 4 presents a world map displaying the geographic distribution of acceptance rates. Interestingly, the map reveals that data for this review was largely sourced from studies conducted in the global north, whereas the global south is poorly represented. China, Canada, France, United Kingdom (UK), and Ireland have the highest acceptance rates between 61% and 80%. They are closely followed by countries such as the United States, Bangladesh, Democratic Republic of Congo, Bulgaria and Greece with acceptance rates between 41% and 60%. Cameroon has the lowest acceptance rate of 15.4%. There is a clear global north dominance with very few studies emanating from continents in the global south such as Africa and South America. This low research output on COVID-19 vaccines uptake in low-income countries of the global south is connected to some of the recurring challenges such as rudimentary laboratories, power cuts, scarce research funding and data sparsity. However, such issues are beyond the scope of this review.

### Review of selected studies

With emerging variants of the COVID-19 pandemic globally including the delta variant, total acceptance of vaccination by healthcare workers is vital as they have a fundamental role in sensitizing patients, advising and directing them to the right clinical decisions. Articles for this review were selected from studies carried out in Asia, Europe, North America and Africa. In a study carried out in Hong Kong by Martin *et al.*, 36 with 1200 participants, the adjusted rate of accepting the SARS-CoV-2 vaccine was 37.2% (95% C.I. 34.5–39.9%). The predicted acceptance rate was highest among the aged than the youth (18–24 years), showing age dependency. A discrete choice experiment was conducted across six Chinese provinces selected by the stratified random sampling method. Vaccine preference sets were structured on seven qualities including: vaccine effectiveness, side effects, number of doses, accessibility, duration of protection, and proportion of contacts vaccinated using conditional logit and latent class models for identifying preferences. Among other investigated attributes, vaccine effectiveness, side effects and percentage of contacts vaccinated were the most important attributes. The researcher also found a higher likelihood of vaccination when the vaccine was more effective; small risks of serious side effects; vaccinations were free and voluntary; fewer doses; a longer duration of protection; and a higher percentage of acquaintances who were vaccinated. Higher local vaccine coverage produces altruistic herd immunity against infection^12^,^45^. The predicted vaccination uptake of the optimal vaccination scenario in their study was 84.77%. Older individuals who had a lower education level, lower income, higher trust in the vaccine and higher perceived risk of infection, showed a higher likelihood to vaccinate 12.

Ruiz and Bell 22 reported a nationwide survey conducted in the United States on predictors of intention to vaccinate against COVID-19. COVID-19 inoculation intentions were weak, with 14.8% of respondents being unlikely to get vaccinated while 23.0% were unsure. The intent to inoculate was highest for men, older people, married or partnered people with pre-existing medical conditions; and those vaccinated against influenza during the 2019–2020 flu season were more motivated towards immunization. In another report, adults with Multiple Sclerosis (N = 486) living in the United States completed a cross-sectional online survey (between 10 April 2020 and 06 May 2020) about their willingness to receive a COVID-19 vaccination once available. Participants also completed measures to describe the sample and to assess factors potentially related to vaccine willingness, including demographics, MS-specific variables, psychological measures, COVID-19 information sources, and perceived trustworthiness of their information sources. It was recorded that approximately two-thirds of the participants (66.0%) reported a willingness to obtain a future COVID-19 vaccine, whereas 15.4% of the sample was unwilling. Greater willingness to receive the vaccine was associated with having a higher level of education and holding a higher perception of one's risk of catching COVID-19. Approximately a third (31.6%) of the sample reported getting their information from healthcare providers. Healthcare providers and the National MS Society had the highest perceived trustworthiness for COVID-19 information. The perceived trustworthiness of information sources was highly associated with vaccine willingness 23.

In a study conducted in the UK to ascertain uncertainty and unwillingness to receive COVID-19 vaccines, a large sample of UK adults (32,361 adults) participated in the study. Four factors were identified to negatively affect SARS-CoV-2 vaccine attitudes: mistrust of vaccine benefits, worries about unforeseen effects, concerns about commercial profiteering, and preference for natural immunity. In a related study on COVID-19 related factors, negative vaccine attitudes, and prior vaccine behavior on uncertainty and unwillingness to be vaccinated for COVID-19, they observed that 16% of respondents demonstrated high levels of mistrust about vaccines. Distrustful attitudes towards vaccination were higher amongst individuals from ethnic minority backgrounds, with lower annual income and poor knowledge of COVID-19. Overall, 14% of respondents reported unwillingness to receive a vaccine for COVID-19, whilst 23% were unsure. The largest predictors of both COVID-19 vaccine uncertainty and refusal were low-income groups (< £16,000, a year), not receiving flu vaccines in the previous year, poor observance of COVID-19 guidelines, female gender, and living with children. Amongst vaccine attitudes, mistrust of vaccine benefits and concerns about imminent unforeseen side effects were the most important determinants of unwillingness to inoculate against COVID-19^33^.

The study by Robertson *et al.*, 34 on ‘Predictors of COVID-19 vaccine hesitancy in the UK Household Longitudinal Study’ using 12,035 participants collected from the ‘Understanding Society’ COVID-19 web survey revealed that the intention to be vaccinated was high (82% likely/very likely). Vaccine hesitancy was higher in women (21.0% vs 14.7%), younger age groups (26.5% in 16 – 24 age bracket vs 4.5% in 75+) and less educated (18.6% no qualifications vs 13.2% degree qualified). Vaccine hesitancy was particularly high in Black (71.8%), Pakistani/Bangladeshi (42.3%), Mixed (32.4%) and non-UK/Irish White (26.4%) ethnic groups. Adjusted models displayed gender, education and ethnicity were associated with COVID-19 vaccine hesitancy. The odds ratios for vaccine hesitancy were 12.96 (95% CI: 7.34, 22.89) in the Black/Black British and 2.31 (95% CI: 1.55, 3.44) in Pakistani/Bangladeshi ethnic groups compared to 3.24 (95% CI:1.93, 5.45) for White British/Irish ethnicity. The main reason for hesitancy was the fear of unknown future effects.

Patelarou *et al.*, 35 also carried out a multicenter cross-sectional study to explore the intention of nursing students to get vaccinated for SARS-CoV-2 infection and the factors acting either as motivators or barriers towards vaccination in 7 countries (Greece, Albania, Cyprus, Spain, Italy, Czech Republic and Kosovo) through a web survey on 2249 nursing students. Forty-four percent of students agreed to accept a safe and effective COVID-19 vaccine, while the acceptance was higher among Italian students. The factors for intention to get vaccinated were male gender (p=0.008), no working experience in healthcare facilities during the pandemic (p=0.001), vaccination for influenza in 2019 and 2020 (p<0.001), trust in doctors (p<0.001), governments and experts (p=0.012), high level of knowledge (p<0.001) and fear of COVID-19 (p<0.001). Understanding the factors that influence students' decisions to accept COVID-19 vaccination could increase the acceptance rate of the vaccines^35^.

In Africa, several studies have been carried out on the acceptance of COVID-19 vaccines. A national survey of potential acceptance of COVID-19 vaccines among healthcare workers was conducted in Egypt by Hussein *et. al.*,^42^. They carried out a cross-sectional online survey that involved 496 healthcare employees; 55% were in the age group of 18-45 years. A history of chronic diseases was recorded in 40.4%, and definite history of drug/food allergy in 10.1%. Only 13.5% totally agreed to receive the vaccine, 32.4% somewhat agreed and 40.9% disagreed to take the vaccine. The disagreements were caused by fears of the safety of the vaccine, fear of genetic mutation and recent techniques and beliefs that the vaccine is not effective (57%, 20.2%, 17.7% and 16.6% respectively). According to their report, the most trusted vaccine was the mRNA-based vaccine. The age of healthcare employees and the presence of comorbidities or chronic diseases were the main factors related to COVID-19 acceptance (p<0.001 and 0.02 respectively), and they concluded that vaccine hesitancy is not uncommon in healthcare employees in Egypt, and this may be an alarming barrier of vaccine acceptance in the rest of the population.

Ditekemena *et al.*, 43 investigated the level of willingness for COVID-19 vaccination in the Democratic Republic of Congo (DRC) through an online survey. A total of 4131 responses were included, and the mean age of respondents was 35 ± 11.5 years. Overall, 2310 (55.9%) indicated they were willing to be vaccinated. In a multivariable regression model, middle and high-income, being tested for COVID-19 and COVID-19 community vaccine acceptance were associated with an increased willingness to be immunized. Being a healthcare worker was associated with a decreased willingness for inoculation^43^.

### Meta-analysis of included studies

**Figure 5:**
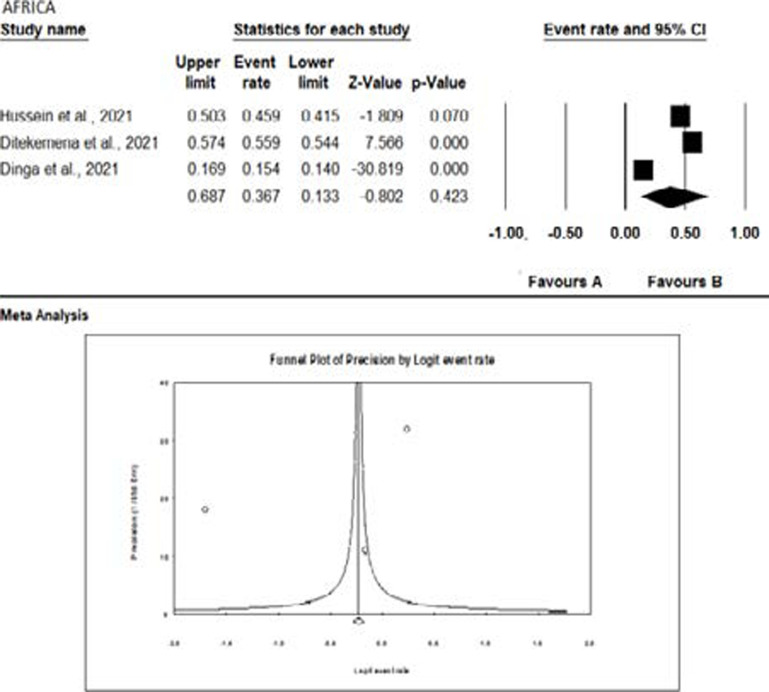
Forest plot of the meta-analysis - Africa

**Figure 6:**
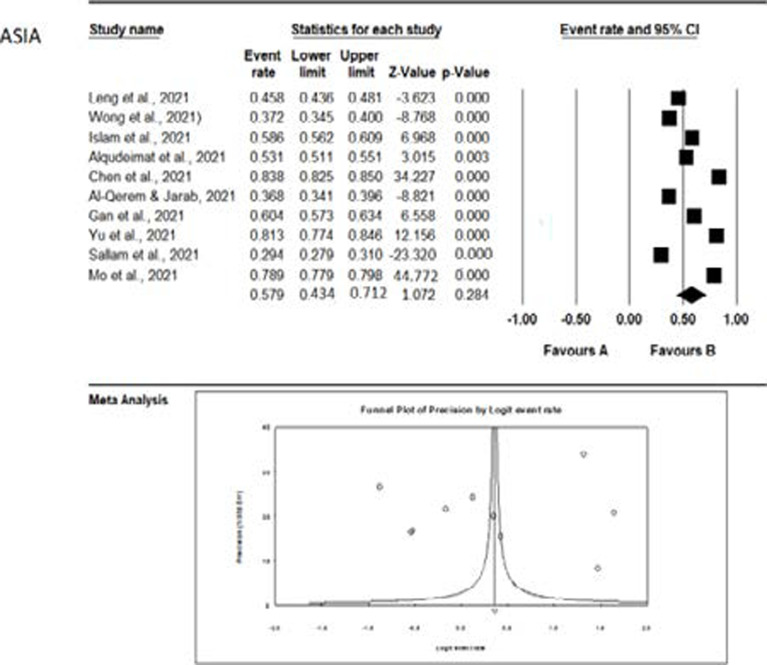
Forest plot of the meta-analysis – Asia

**Figure 7:**
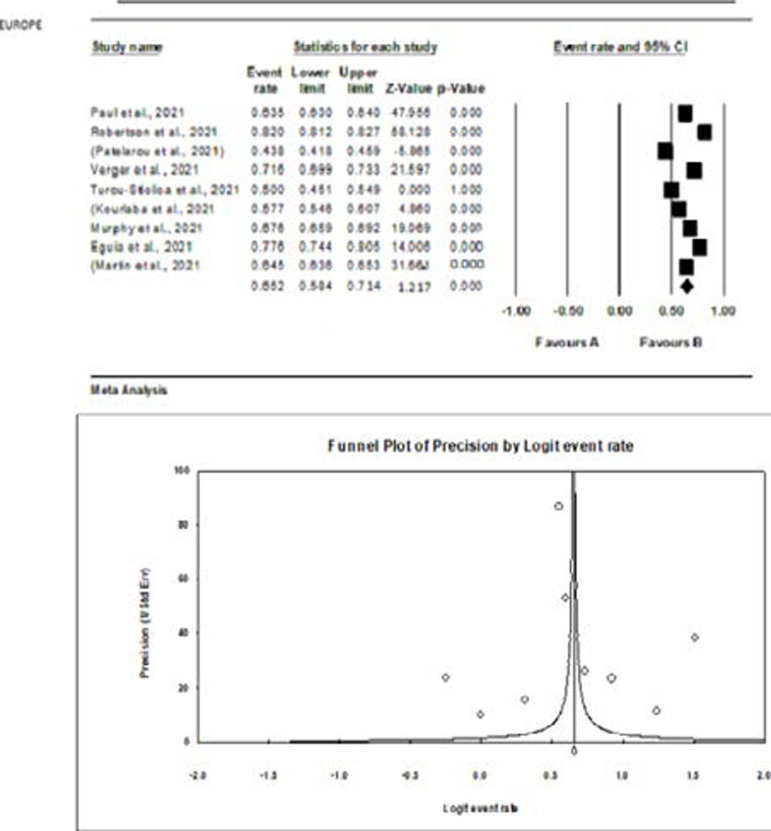
Forest plot of the meta-analysis – Europe

**Figure 8:**
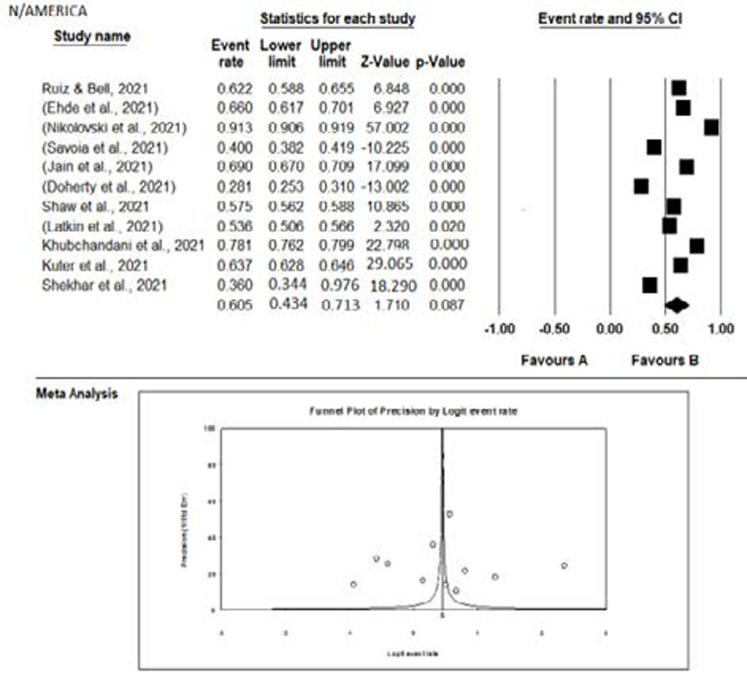
Forest plot of the meta-analysis – North America

**Figure 9:**
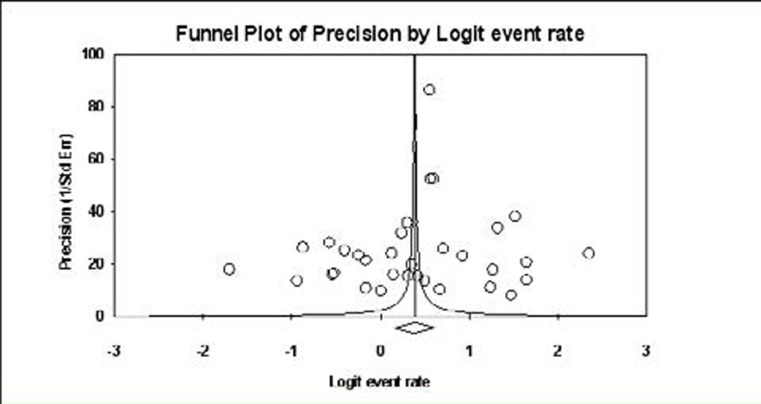
Summary funnel plot for publication bias showing the level of moderate precision with less publication bias

The meta-analysis of the studies from the different continents is graphically represented with the aid of forest plots in Figures 5 – 9. Also shown in the figures are the citations and the respective statistics of the studies included in the meta-analysis. While Figures 5 - 8 show the forest plots for the four continents, (data was not obtained for the other continents), Figure 9 shows a summary funnel plot for publication bias.

In the studies from Africa (Figure 5), the pooled effect is significant and not due to chance with an acceptance rate of 36.7%.

In the studies from Asia (Figure 6), a high acceptance rate (57.9%) was observed. However, this is not statistically significant and could be influenced by chance.

In the studies from Europe (Figure 7), a high acceptance rate (65.2%) was observed which was statistically significant and is not influenced by chance.

In the studies from North America (Figure 8), a high acceptance rate (60.5%) was observed but was not statistically significant and could be influenced by chance.

Table 2 shows the general statistics for the meta-analysis (95% CI).

**Table 2:** The general statistics for the meta-analysis (95% CI)

**Effect size and 95% interval**			

Measure	Value	Lower limit	Upper limit
Point estimate	0.623320	0.62054000	0.62609230

**Test of null (2-Tail)**			

Measure	Z-value	P-value	
	83.4884038	0.000	
	3.16554698	0.00154792	

	**Heterogeneity test**		

**Measure**	**Value**		

Q-value df	11859.137147626		
(Q)	32		
P-value	0.000		
I-squared	99.7301658661869		

	**Tau-squared**		


**Measure**	**Value**		

Tau Squared	0.48337936120		
Standard Error	0.2112679534		
Variance	4.4634148E-02		
Tau	0.695254889		

## Discussion

In this systematic review and meta-analysis involving 33 eligible articles, we assessed the acceptance rates of COVID-19 vaccines in a general population of 123,282 individuals (including healthcare workers) globally.

### Key findings

The meta-analysis revealed a significant difference between the willingness of the population to be vaccinated and those that were not willing (Appendix 1). Also, low COVID-19 vaccine acceptance rates below 50% were not specifically clustered in a particular region of the world. Consequently, Europe, North America, Africa and Asia all show low acceptance rates in different populations (45.82% in China; 37.2% in Hong Kong; 45.9% in Egypt; 40% in the US, 43.8% in Greece, Albania, Cyprus, Spain, Italy, Czech Republic and Kosovo; 28.1% in the US (North Carolina); 36.8% in Jordan; 29.4% in Jordan, Kuwait and Saudi Arabia, 36% in the US and 15.4% in Cameroon.

The continental evaluation revealed variations between continents and a lack of data emanating from certain continents. The pooled analysis revealed 36.7% (CI; 13-69%) for Africa, 57.9% (CI; 43.4-71.2) for Asia, 65% (CI; 58.4-71.4%) for Europe and 61% (CI; 34.4-71.3%) for North America. The observed lowest willingness to accept the vaccine in Africa was 36.7% compared to Europe (65%), North America (61%) and Asia (57.9%). The low-level willingness in Africa could be based on reported factors including fear of side effects, the lack of trust in the vaccines, and possibly the lower health risk of the pandemic in the region compared to other parts of the globe. The pooled effect was however only statistically significant for Europe (p < 0.05) shown in Figure 7. This observation suggests that the observed high willingness for vaccination among the European populace was not accidental. The high willingness in Europe confirms the high cases and mortality in the region during the study and hence the willingness to vaccinate. The perceived risk is therefore a key factor in vaccine uptake as observed in the vast difference between Arica and Europe in the present study.

Interestingly, healthcare personnel who are presumably at the frontline of the coronavirus and with higher risk of infections report varying acceptance rates in these studies. Of the 38 studies, four out of nine studies involving healthcare professionals or students had acceptance rates ≤ 50% 42 35 38 32. From the geographical distribution of the acceptance rates (Figure 4), the global south showed a lower acceptance rate compared to the north.

### Comments

Healthcare workers (HCW) and the elderly are at higher risk of COVID-19 and ideally, are expected to have high acceptance rates of COVID-19 vaccines. The HCWs could also be advocates to promote vaccine acceptance within their societies. Research has shown that healthcare workers (HCWs) who accept vaccines would recommend such to their loved ones and patients 46,47,48,49. However, in the included studies in this review, the highest acceptance rate among HCW was 71.6% 37; lower than the 90% in an elderly US population 24. The difference in acceptance rates in the global north and south could be due to limited data or limited research on the acceptance rates of vaccines in the south, or perhaps, due to the survey methods used among countries in the global south. Nevertheless, there is a need for continuous updates to be made available, especially since healthcare workers may depend on scientific publications to update their knowledge and boost their confidence in accepting and promoting COVID-19 vaccination.

The number of people that need to be immunized to achieve herd immunity is not known for COVID-19 50, however, efforts to increase vaccination and address the challenges in the general population are necessary. Two variables that may be considered from the theoretical model of Rosenstock's Health Belief Model (HBM) 51,52 in relation to COVID-19 vaccination are perceived barriers to accepting vaccination and the perceived benefits of accepting vaccination. Addressing these barriers and promoting the vaccine benefits through awareness and sensitization may enlighten individuals to understand the more positive outcomes and lesser negative consequences associated with COVID-19 vaccination. In this current pandemic, vaccine acceptance may be a strong factor to control the situation. As the large-scale production of vaccines to meet up global demand may be challenging, people who can access the vaccine should consider it a privilege and accept it to ensure effective control of the pandemic.

In our review, Cameroon, a developing country, recorded the lowest acceptance rate of 15.4% 44. Similarly, a low acceptance rate was also visible in a developed society - North Carolina, US 27. This could suggest that different underlying factors are present in different societies that could affect vaccine acceptability, irrespective of country or regional classification. Thus, there is need a for citizens to gain confidence in their government, with local measures considered for educating, sensitizing and promoting COVID -19 vaccination programs. Thus, negative political influences on vaccination should be eliminated while ensuring wider coverage to targeted populations.

### Strengths and limitations

The strength of this review is in the systematic search of available literature using different databases. However, there are some limitations in the review, and this should be considered while interpreting the results. The responses used to induce the acceptance rates varied across studies, for example, ‘Agree to vaccinate’, ‘extremely or somewhat likely’, ‘Yes/No’, and ‘Soonest/after clarification’. The cross-sectional design of studies as well as some studies involving more than one country in their surveys can affect the true interpretation of the acceptance rates regionally. Finally, the studies were not homogenous in their population groups, thus, it may not apply to all population groups or workers and might have contributed to the high heterogeneity.

### Implications for practice and the future

This review can be a guide to target specific populations or regions as well as a motivation towards accepting COVID-19 vaccination to control the pandemic. The low acceptance rates among healthcare workers or students needs to be improved, as these are the professionals that will contribute to enlightening the public on the importance of COVD-19 vaccination. In the future, vaccination programs should seek to address fears over unknown future vaccine effects, fear of genetic mutation and distrustful attitudes towards vaccination. Individuals from ethnic minority backgrounds and those in lower-income groups should not be neglected.

## Conclusion

The COVID-19 pandemic remains, with evolving variants and a continual increase in the number of cases across the globe. The breakthrough in the development of COVID-19 vaccines is a step towards curtailing the burden of COVID-19. However, wide variations in the acceptance of the vaccines poses a major challenge to achieving herd immunity through vaccination. Most of the studies reported COVID-19 vaccine acceptance rates below 70%. This should be a point of concern especially as previous vaccination programs for measles and polio required a threshold of 95% and 80% vaccination to achieve herd immunity 50.

In summary, some factors including vaccine effectiveness, side effects being a male, being a white, married, being in the high-risk group, prior vaccination with influenza, lower income, higher trust in the vaccine and higher perceived risk of infection, perceived trustworthiness of information sources were observed to positively influence COVID-19 vaccine uptake.

## Data Availability

Supplementary data has been attached.
